# Estimation of economic injury levels and action thresholds for the two-spotted spider mite, *Tetranychus urticae* Koch, on strawberry plants

**DOI:** 10.1007/s10493-026-01159-2

**Published:** 2026-08-19

**Authors:** Eman M. Abdelmaksoud, Shoukry A. El-Refai, Kadry W. Mahmoud, Walaa El-Sayed, Mohamed E. Ragab

**Affiliations:** 1 Department of Plant Protection, Faculty of Agriculture, Ain Shams University, P.O. Box 68, Hadayek Shubra, Cairo, 11241 Egypt; 2 Department of Horticulture, Faculty of Agriculture, Ain Shams University, P.O. Box 68, Hadayek Shoubra, Cairo, 11241 Egypt

**Keywords:** Acari, Tetranychidae, Pest management, Crop loss assessment, Economic thresholds, Injury level

## Abstract

Strawberry (*Fragaria × ananassa* Duch.) is a high-value global crop, yet its productivity is severely constrained by the two-spotted spider mite, *Tetranychus urticae* Koch. Developing localized economic thresholds is a cornerstone of sustainable Integrated Pest Management (IPM) to mitigate yield losses and reduce acaricide reliance. This two-season field study (2019–2020) investigated the quantitative relationship between *T. urticae* population density and strawberry yield in Egypt. Using varying infestation levels established via Chitosan (Top9 0.1%) treatments, linear regression and Chi-square (χ^2^) analyses were employed to determine four critical economic levels: General Equilibrium Position (GEP), Economic Threshold Level (ETL), Economic Injury Level (EIL), and Economic Damage Level (EDL). Results revealed a highly significant negative correlation between mite density and yield (*r* = -0.96*** in 2019; *r* = -0.97*** in 2020), with an average yield reduction of 1.25% per mite. The estimated EIL and ETL were 4.0 mites/leaf in 2019 and 3.4 mites/leaf in 2020. These findings provide a precise decision-making framework for mite management in Mediterranean strawberry production. The low estimated thresholds suggest that proactive, preventive management strategies are more effective than reactive control to maintain strawberry yield.

## Introduction

Strawberry (*Fragaria × ananassa* Duch.) is one of the most economically important fruit crops worldwide and is widely cultivated under both open-field and protected conditions. The crop is valued for its high nutritional content, pleasant flavor, and substantial economic return to growers (Hannum 2004). Egypt is a leading exporter of strawberries, particularly frozen strawberries, capturing approximately 27% of the global market in 2024 with export volumes of 353,000 tons and export value of around USD 383 million (Amwal Al Ghad 2025). In 2025, export revenues surged to USD 697 million, highlighting the crop’s significant contribution to the national economy (Egyptian Gazette 2026). Among these, the two-spotted spider mite, *Tetranychus urticae* Koch (Acari: Tetranychidae), is considered one of the most destructive pests of strawberries in many production regions (El-Saiedy and Fahim 2021; Zhao et al. 2023). This highly polyphagous mite attacks over 1,000 plant species, feeding primarily on the lower leaf surfaces by piercing epidermal cells and sucking their contents. Infestation leads to chlorotic stippling, leaf bronzing, reduced photosynthetic activity, premature leaf senescence, and substantial decreases in plant vigor and productivity (Parmagnani et al. 2023). Field studies have demonstrated that increasing population densities of *T. urticae* are strongly associated with reductions in marketable strawberry yield. For instance, significant crop losses and yield reductions are triggered when mite populations reach specific economic thresholds, ranging from 50 to 80 motile mites per leaf depending on seasonal and environmental variations (Nyoike and Liburd 2013). Despite these quantified findings, evaluating these thresholds locally remains essential due to regional differences in agricultural practices and climate.

The rapid population density increase of *T. urticae*, attributed to its short life cycle and high reproductive capacity, particularly under favorable conditions such as high temperature and low humidity, coupled with its notorious ability to rapidly develop resistance to acaricides (Alpkent et al. 2025), often results in control failures in strawberry production. Consequently, early detection and accurate monitoring of mite populations are essential components of integrated pest management (IPM) programs. Timely determination of population levels at which economic damage occurs is crucial for developing efficient management strategies and minimizing unnecessary pesticide applications (Choi et al. 2022).

The concept of economic thresholds is a cornerstone of modern integrated pest management (IPM) programs. The economic threshold level (ETL) represents the pest population density at which control measures should be implemented to prevent the pest population from reaching the economic injury level (EIL), which is the point where the cost of pest damage equals the cost of control (Pedigo et al. 1986). Estimating these economic levels allows growers to apply control measures judiciously, reducing unnecessary pesticide applications and minimizing production costs (Pedigo et al. 1986). In the case of *T. urticae*, its rapid population growth and high reproductive potential make the establishment of these thresholds particularly critical. Field studies on strawberries have demonstrated that increasing mite densities correlate strongly with reductions in marketable yield, providing quantitative data to establish ETL and EIL for practical pest management (Nyoike and Liburd 2013). Despite the abundance of biological and control studies, local evaluation of these economic levels under Egyptian environmental conditions remains limited, highlighting the need for region-specific assessments to guide sustainable IPM strategies.

Consequently, the present study sought to quantify the dynamic relationship between *T. urticae* density and strawberry yield loss under Egyptian field conditions. The primary objective was to establish reliable economic parameters, specifically the General Equilibrium Position (GEP), Economic Threshold Level (ETL), Economic Injury Level (EIL), and Economic Damage Level (EDL). By tailoring these thresholds to the local environment, this research provides a strategic framework for sustainable IPM, ensuring that intervention is both economically justified and environmentally responsible.

## Materials and methods

### Experimental design and field setup

This experiment was conducted over two consecutive seasons (2019 and 2020) at a farm located in Elatta, Giza governorate, Egypt (30°01’55” N, 31°06’38” E). In both seasons, strawberry plants of the ‘Festival’ cultivar were planted in mid-September and maintained until the end of April. The experimental area, approximately 315 m², was divided into seven equal plots, each measuring about 45 m². Each plot was further subdivided into three replicates, with each replicate spanning approximately 15 m². The *T. urticae* populations evaluated in this research originated entirely from indigenous, natural field infestations occurring spontaneously on the strawberry plants. Prior to the study, species identification for *T. urticae* was morphologically verified and confirmed via microscopic examination using standard taxonomic keys. To establish varying levels of *T. urticae* infestation, strawberry plants were subjected to different spraying schedules using Top9 (Chitosan 0.1%) at a rate of 1000 cm³/Fadden. The insecticidal formulations were applied using a compression knapsack sprayer. Spraying commenced on March 9th in both seasons, coinciding with the peak fruiting and harvesting stage, during which marketable fruits were periodically harvested and weighed to evaluate yield loss. Treatments were applied according to the following treatment schedule (Table 1):

**Table 1:** Number and dates of Top9 (acaricide) applications across different treatment intervals against *T. urticae* on strawberry plants at Giza Governorate during the 2019 season

Treatment No.	I	II	III	IV	V	VI	VII
Spray interval	10-day interval	12-day interval	15-day interval	18-day interval	28-day interval	60-day interval	Control
1	9 Mar	9 Mar	9 Mar	9 Mar	9 Mar	9 Mar	-
2	19 Mar	21 Mar	23 Mar	27 Mar	6 April	-	-
3	29 Mar	2 April	6 April	13 April	-	-	-
4	8 April	14 April	20 April	-	-	-	-
5	18 April	25 April	-	-	-	-	-
6	28 April	-	-	-	-	-	-

Treatment I: Plants received 6 sprays at 10-day intervals.

Treatment II: Plants received 5 sprays at 12-day intervals.

Treatment III: Plants received 4 sprays at 15-day intervals.

Treatment IV: Plants received 3 sprays at 18-day intervals.

Treatment V: Plants received 2 sprays at 28-day intervals.

Treatment VI: Plants received 1 spray at 60-day intervals.

Treatment VII: Control plants, left untreated.

All experimental plots received standard agricultural practices throughout the study period.

### *T. urticae* infestation and yield assessment

Infestation levels of *T. urticae* were estimated by collecting samples of 10 randomly selected leaves from the 50 plants within each replicate at 7-day intervals, specifically selecting mature, fully expanded leaves from the middle tier (stratum) of the plant canopy to ensure sample homogeneity, starting from March 9th until the end of April, extending through the harvest period. The collected leaves were placed in paper bags and transported to the laboratory for examination and counting of only the motile stages (larvae, nymphs, and adults) of the *T. urticae* under a stereomicroscope. Strawberry fruits were harvested throughout the harvest season in both 2019 and 2020. The total weight of fruits produced by 50 plants in each treatment was recorded, and the average yield weight was calculated.

### Statistical analysis and economic level estimation

To assess the relationship between *T. urticae* infestation and strawberry fruit production, simple correlation and linear regression analyses were applied. The population density of the movable stages of *T. urticae* was considered the independent variable (x), while the strawberry fruit production was the dependent variable (y). The linear regression formula, (Ŷ) = a + bX (Goulden 1960), was used to detect variations in fruit production due to infestation. Here, Ŷ represents the estimated production weights, X is the degree of infestation, ‘a’ is the intercept, and ‘b’ is the slope, representing the decrease in ‘y’ for a unit increase in ‘x’. The linear regression coefficient ‘b’ was calculated when the simple correlation coefficient ‘r’ was significant. Additionally, curvilinear nonlinear regression was estimated, and the coefficient of determination (r²) was calculated. For testing the homogeneity of control agents, the Chi-square (χ²) method was employed (Snedecor and Cochran 1987), using the formula:

$$\chi^2 = \frac{{\sum \left( {ai*pi} \right) - C1*\bar Pi}}{{\bar Pi*\bar qi}}$$

where ¯Pi = C1 / G and ¯q = 1 - ¯Pi.

To quantify the production reduction caused by a single *T. urticae*, the following equation was applied:

$${\% \mathrm{Reduction}} = \frac{b}{{\;\mathop y\limits^ - }} \times 100$$

where ‘b’ is the unit effect (regression value) and ȳ is the average mean production per sample. Estimation of economic levels of infestation, including the General Equilibrium Position (GEP), Economic Threshold Level (ETL), Economic Injury Level (EIL), and Economic Damage Level (EDL), was based on the correlation between different levels of *T. urticae* population density and strawberry fruit production per plant. These terms are defined as follows, according to Pedigo et al. (1986).

The economic levels of *T. urticae* on strawberry plants were estimated by converting the mean mite counts into “mites per leaf” to establish precise thresholds for pest management. To determine the approximate damage threshold, an attempt was made to identify the point on the curved regression slope where fruit production began to show a significant drop. The infestation level corresponding to this point was considered the Damage Threshold, necessitating immediate control measures to prevent significant production reduction. This was achieved using Chi-square analysis (χ²) or “rx²” contingency tables, assuming a total of 50 strawberry plants (R) producing fruit. To contextualize the pest population dynamics, meteorological data (including daily temperature and relative humidity) during the 2019 and 2020 experimental seasons were monitored and obtained from the Central Laboratory for Agricultural Climate (CLAC), Agricultural Research Center, Giza, Egypt.

## Results

### Infestation- fruit production relationship

Statistical analysis revealed that the varying acaricide spray intervals and frequencies (detailed in Table 1) directly governed the seasonal mean density of *T. urticae*. Shorter spray intervals coupled with higher application frequencies progressively suppressed mite population development. Specifically, Treatment I (10-day interval, 6 sprays) maintained the lowest mean *T. urticae* density (11.9 and 6.88 mites/10 leaves) and achieved the highest marketable strawberry crop yield (8.7 and 6.7 Kg/50 plants) in both the 2019 and 2020 seasons, respectively. Conversely, wider spray intervals allowed higher *T. urticae* buildup, culminating in the highest density (56.0 and 53.26 mites/10 leaves) and lowest yield (4.0 and 5.0 Kg/50 plants) within the untreated control (Treatment VII).

**Fig. 1:**
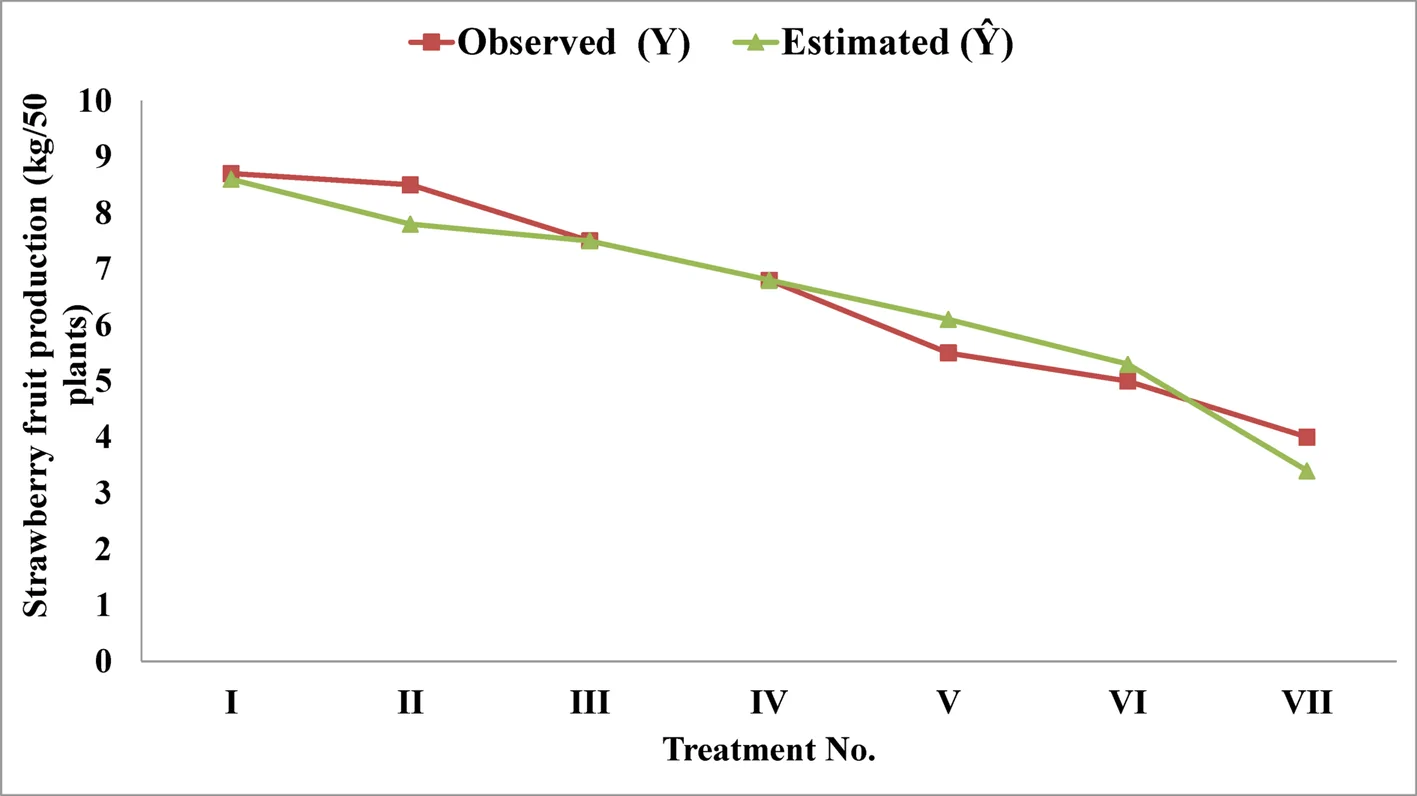
Validation of the linear regression model for the 2019 season: Comparison between observed (Y) and estimated (Ŷ) strawberry fruit production (kg/50 plants) across seven treatment levels of *T. urticae* infestation

**Fig. 2:**
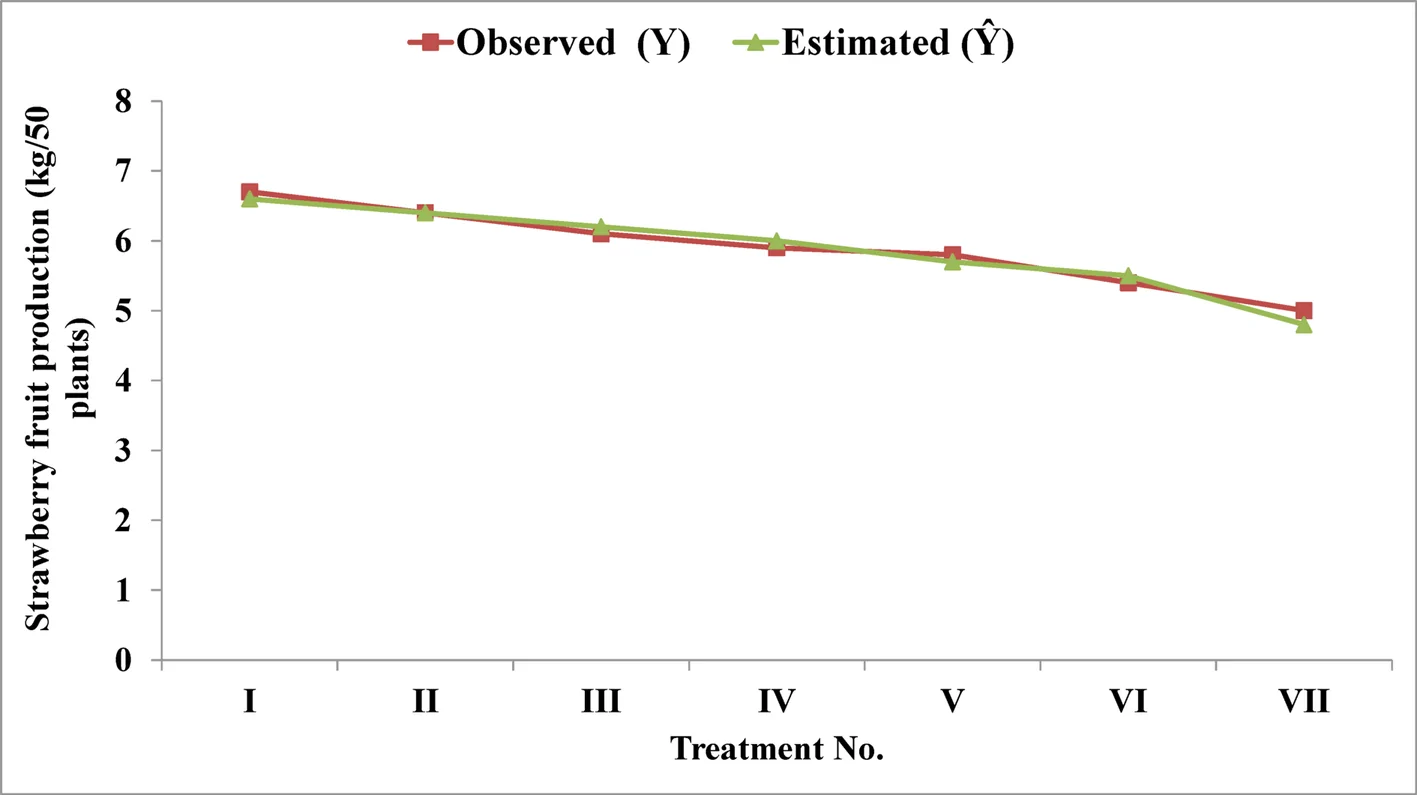
Validation of the linear regression model for the 2020 season: Comparison between observed (Y) and estimated (Ŷ) strawberry fruit production (kg/50 plants) across seven treatment levels of *T. urticae* infestation

Consequently, a highly significant negative correlation was found between *T. urticae* population density and strawberry fruit production across both experimental seasons (Table 2). In the 2019 season, the correlation coefficient (*r*) was − 0.96***, and the regression coefficient (*b*) was − 0.117 kg/mite. Similarly, for the 2020 season, the correlation coefficient (*r*) was − 0.97***, with a regression coefficient (*b*) of -0.04 kg/mite. The coefficients of determination (*r²*) were 0.92 and 0.94 for the 2019 and 2020 seasons, respectively, indicating that a substantial proportion of the variation in strawberry fruit production could be explained by *T. urticae* population density (Table 2). The estimated crop yield (Ŷ) was calculated using the prediction equation: Ŷ = ȳ ± b(x - x̄) (Figs. 1 and 2). Table 3 presents the comparison between observed (Y) and estimated (Ŷ) fruit production data for both seasons. Further analysis indicated that a single individual of *T. urticae* caused a fruit production reduction of 1.8% in the 2019 season and 0.7% in the 2020 season, resulting in an average reduction of approximately 1.25% in total fruit production per mite across the study period.

**Table 2:** Correlation and regression analysis between *T. urticae* population density and strawberry fruit production during the 2019 and 2020 seasons

Treat. No.	2019 season		2020 season	
	Mean number of T. urticae/10 leaves	Fruit production in Kg/50 plants	Mean number of T. urticae/10 leaves	Fruit production in Kg/50 plants
I	11.9	8.7	6.88	6.7
II	18.5	8.5	13.3	6.4
III	21.8	7.5	16.4	6.1
IV	27.6	6.8	22.22	5.9
V	32.9	5.5	28.9	5.8
VI	40	5	34.42	5.4
VII	56	4	53.26	5
Total	208.7	46	175.38	41.3
Mean	29.8	6.5	25	5.9
*r*	-0.96***		-0.97***	
*b*	-0.117		-0.04	
*r* ^*2*^	0.92		0.94	

*Abbreviations*: ***r***: Simple correlation coefficient. ***b***: Regression coefficient (Slope). ***r²***: Coefficient of determination

***: Highly significant at 0.001

**Table 3:** Observed (Y) and estimated ($\:\widehat{\mathrm{Y}\:}$) strawberry fruit production (kg/50 plants) based on *T. urticae* population density during the 2019 and 2020 seasons

Treatment No.	2019		2020	
	Observed (Y)	Estimated (Ŷ)	Observed (Y)	Estimated (Ŷ)
I	8.7	8.6	6.7	6.6
II	8.5	7.8	6.4	6.4
III	7.5	7.5	6.1	6.2
IV	6.8	6.8	5.9	6
V	5.5	6.1	5.8	5.7
VI	5	5.3	5.4	5.5
VII	4	3.4	5	4.8

(Y)= Observed fruit production data (kg/50 plants). (Ŷ)= Estimated fruit production data derived from the linear regression model

The close proximity between (Y) and (Ŷ) values confirms the high reliability of the regression models (r^2^ ≥0.92)

### Estimation of economic levels of infestation

#### Season 2019 (Table 4)

The impact of *T. urticae* density on yield was highly significant (**χ**^**2**^ **=** 44.18, P˂0.001). Based on the production reduction analysis, four economic levels were identified:

**Table 4:** Chi square (*χ*^*2*^) analysis for the impact of *T. urticae* population density on the fruit production of strawberry var. Festival at Giza governorates during the 2019 season

No. of treatment	*N*	a_i_	*R*	*P*_i_(a_i_/*R*)	a_i_*P_i_	Groups
I	8.7	11.9	50	0.238	2.8322	G.E.P.=2.76
II	8.5	18.5	50	0.37	6.845	
III	7.5	21.8	50	0.436	9.5048	
IV	6.8	27.6	50	0.552	15.2352	
V	5.5	32.9	50	0.658	21.6482	E.T.L.= 4
VI	5	40	50	0.8	32	
VII	4	56	50	1.12	62.72	E.I.L.=5.6
Total	46	C_1_=208.7	G = 350	P_i_ =4.17	∑ a_i_P_i_=150.7	

Calculated *χ*^*2*^ = 44.18*** (Highly significant at P˂0.001), Tabulated *χ*^*2*^ at degree of freedom n-1 and a significance level at 0.001 = 22.46)

N= Fruit production in Kg/50 plant, ai= Mean number of *T. urticae*/ 10 leaves, R= Number of plants (50). G.E.P.= General Equilibrium Position; E.T.L.= Economic Threshold Level; E.I.L.= Economic Injury Level

##### General Equilibrium Position (GEP)

Established at 2.76 mites/leaf. At this level, the population is in equilibrium and does not cause significant economic impact.

##### Economic Threshold Level (ETL)

Observed at 4.0 mites/leaf. This level serves as the “action threshold” where control measures should be initiated to prevent the population from reaching the injury level (χ²=10.7, P˂0.01).

##### Economic Injury Level (EIL)

Identified during the population increase between 2.76 and 4.0 mites/leaf. In this range, the fruit production loss began to justify the costs of intervention (χ²=7, P˂0.05).

##### Economic Damage Level (EDL)

Reached when the density rose to 5.6 mites/leaf. This level recorded the maximum significant fruit production loss (reduced to 4.0 kg/50 plants), with a high partial Chi-square value (χ²=66.6, P˂0.001).

#### Season 2020 (Table 5)

For the 2020 season, the mite population showed a more severe impact on yield (**χ**^**2**^ **=** 57.85, P˂0.001). The thresholds were lower than the previous season:

**Table 5:** Chi square (*χ*^*2*^) analysis for the impact of *T. urticae* population density on the fruit production of strawberry var. Festival at Giza governorates during the 2020 season

No. of treatment	*N*	a_i_	*R*	Pi (a_i_/*R*)	a_i_*P_i_	Groups
I	6.7	6.88	50	0.1367	0.94	G.E.P.=2.22
II	6.4	13.30	50	0.266	3.53	
III	6.1	16.40	50	0.328	5.37	
IV	5.9	22.22	50	0.4444	9.87	
V	5.8	28.90	50	0.578	16.70	E.T.L.= 3.4
VI	5.4	34.42	50	0.6884	23.69	
VII	5.0	53.26	50	1.0652	56.73	E.I.L.=5.3
Total	41.3	C_1_=175.38	G = 350	P_i_ =3.50	∑a_i_P_i_=116.87	

Calculated *χ*^*2*^ = 57.85*** (Highly significant at P˂0.001), Tabulated *χ*^*2*^ at degree of freedom n-1 and a significance level at 0.001 = 22.46)

N= Fruit production in Kg/50 plant, ai= Mean number of *T. urticae*/ 10 leaves, R= Number of plants (50). G.E.P.= General Equilibrium Position; E.T.L.= Economic Threshold Level; E.I.L.= Economic Injury Level

##### General Equilibrium Position (GEP)

Identified at 2.22 mites/leaf.

##### Economic Threshold Level (ETL)

Observed at 3.4 mites/leaf, where a significant fruit production drop was recorded (χ²=11.8, P˂0.01).

##### Economic Injury Level (EIL)

Reached as the density increased toward the threshold, with a fruit production loss contribution of 0.6 kg (χ²=6.29, P˂0.05).

##### Economic Damage Level (EDL)

Confirmed at 5.3 mites/leaf. At this density, the fruit production reached its lowest level (5.0 kg/50 plants), justifying intensive control measures (**χ**^**2**^ **=** 32.6, P˂0.001).

## Discussion

The results of the present study demonstrated a strong negative relationship between the population density of *T. urticae* and strawberry fruit production during both experimental seasons. The highly significant correlation coefficients (*r* = − 0.96 and − 0.97) indicate that increases in mite population density were closely associated with reductions in marketable fruit production weights. These findings are consistent with previous studies reporting that infestations of *T. urticae* can substantially reduce strawberry productivity when populations reach moderate to high densities (Nyoike and Liburd 2013; Choi et al. 2022).

The estimated economic thresholds obtained in this study were relatively low, with ETL values of approximately 3.44–4.0 motile stages per leaf (equivalent to about 34–40 individuals per 10 leaves). Such relatively low threshold values can be explained by the biological characteristics of *T. urticae*, which is known for its extremely short life cycle and high reproductive potential. These traits allow mite populations to increase rapidly within a short period of time, particularly under warm and dry environmental conditions that favor their development.

In addition to its rapid reproductive capacity, *T. urticae* possesses remarkable adaptive abilities that facilitate its rapid establishment on new host plants. Experimental evolution studies demonstrated that spider mite populations can develop efficient detoxification mechanisms and adapt to novel plant hosts within approximately 25 generations, highlighting the extraordinary ecological plasticity of this pest (Salehipourshirazi et al. 2021). Furthermore, the arrhenotokous reproductive system and high fecundity of the species accelerate population growth and contribute to the rapid evolution of resistance to acaricides (Van Leeuwen et al. 2010). In North African regions, *T. urticae* may complete up to 27 generations per year under favorable environmental conditions (Assouguem et al. 2022), which further increases the risk of sudden population outbreaks.

The low density and relatively conservative economic thresholds observed in our experimental plots are dynamic field metrics directly governed by local environmental conditions, the targeted scheduling and protective effects of our acaricide treatments, and cultural practices during the harvest window. Because of this rapid population growth, even relatively low initial densities may quickly develop into damaging infestations. In practical field conditions, a small number of mites present on leaves may multiply rapidly within a few days, reaching levels capable of causing significant physiological stress to strawberry plants. Therefore, waiting until visible population build-up occurs may allow the *T. urticae* population to exceed the economic injury level before effective control measures can be implemented. This phenomenon highlights the importance of early monitoring and preventive intervention as essential components of integrated pest management (IPM) programs targeting *T. urticae* (Alpkent et al. 2025).

A comparative analysis of economic thresholds reported in the literature reveals considerable variation among crops, geographic regions, and production systems. The ETL obtained in the present study (3.44–4.0 individuals leaf⁻¹) is substantially lower than that reported for strawberry in Florida, USA, where an ETL of 50 individuals per leaf and an EIL of 80 individuals per leaf were estimated (Nyoike and Liburd 2013). Such regional variation may be attributed to climatic differences, as warm and dry environmental conditions commonly observed in Egypt can accelerate mite development and population growth. Similar relatively low thresholds were reported in Egyptian wheat crops, where ETL values ranged from 4 to 10 individuals per leaf (Kalmosh 2018).

Host plant characteristics may also contribute to variations in economic thresholds among cropping systems. Herbaceous crops such as strawberry, wheat, and tomato generally exhibit greater physiological sensitivity to mite feeding compared with woody perennial crops. For instance, relatively low thresholds have been reported in greenhouse tomato, where the ETL ranged from 1 to 2 individuals per leaflet and the EIL ranged from 5 to 10 individuals per leaflet (Jayasinghe and Mallik 2011). In contrast, higher thresholds have been observed in woody perennial crops such as clementine orchards, where ETL values ranged from 10 to 15 individuals m⁻² of leaf area and the EIL reached 31.1 individuals m⁻² (Pascual-Ruiz et al. 2014). This difference likely reflects the greater structural resilience and longevity of perennial leaves compared with the more delicate tissues of herbaceous crops, which are more susceptible to rapid chlorophyll depletion and desiccation caused by mite feeding (Parmagnani et al. 2023).

Economic thresholds may also vary depending on crop phenology and seasonal conditions. For example, in greenhouse cucumber, Park and Lee (2007) reported that the economic injury level of *T. urticae* fluctuates during the growing season and may reach its lowest values during periods of high crop sensitivity, particularly in spring. Similarly, in soybean, Padilha et al. (2020) indicated that economic thresholds can also be defined using plant damage symptoms rather than mite density alone, with intervention recommended when early yellow mottling symptoms appear and densities approach approximately one individual per cm² of leaf area.

### Limitations of the Work and Future Recommendations

Despite extensive research on *T. urticae* in different cropping systems, quantitative economic thresholds for *T. urticae* under Egyptian strawberry production conditions remain poorly documented. Therefore, the present study provides one of the first field-based estimations of the economic threshold for *T. urticae* in strawberry under Egyptian agro-climatic conditions. However, certain limitations of this work must be fully explained to guide future indexing models. First, our EIL/ETL equations rely strictly on numerical motile densities rather than a formalized visual leaf damage index. Biologically, host plant tolerance fluctuates based on seasonal vigor, meaning it is entirely possible to observe multiple individuals on a leaf that shows no visual symptoms, or conversely, leaves with few individuals exhibiting high levels of damage. Second, field evaluations excluded egg population carry-over dynamics and were calculated based on fixed acaricide and market pricing specific to the study period. Future regional research should integrate automated visual damage scoring and multi-year market fluctuations to further refine dynamic IPM frameworks. The relatively low thresholds identified in this study highlight the importance of proactive pest management strategies in strawberry production systems. Early monitoring, threshold-based decision making, and the integration of biological and chemical control measures are essential for maintaining mite populations below economically damaging levels and ensuring sustainable strawberry production under North African conditions.

## Conclusion

The findings of this two-season study underscore the high sensitivity of strawberry plants to *T. urticae* infestation. The results demonstrated that even a minimal population density (as low as 2.22 to 2.76 mites/leaf) can establish the General Equilibrium Position (GEP), beyond which yield losses begin to accumulate rapidly. With an average yield reduction of 1.25% per mite individual, the Economic Threshold Level (ETL) was identified at a remarkably low range (3.4 to 4.0 mites/leaf).

This narrow margin between the GEP and the Economic Injury Level (EIL) highlights the critical importance of preventive control strategies. Relying on reactive curative measures once infestation is visually apparent may lead to irreversible economic damage, as the Economic Damage Level (EDL) is reached at densities as low as 5.3 to 5.6 mites/leaf. Therefore, to optimize strawberry yield and quality in Egypt, Integrated Pest Management (IPM) programs should prioritize early-season monitoring and proactive intervention (such as the use of Chitosan-based products like Top9) before mite populations escalate. These localized thresholds provide a precise roadmap for growers to minimize pesticide reliance while maintaining economic sustainability.

## Data Availability

The datasets generated during and/or analyzed during the current study are available from the corresponding author on reasonable request.
